# In Vitro Effectiveness of Endodontic Triple Antibiotic Paste Associated With Daptomycin

**DOI:** 10.7759/cureus.90108

**Published:** 2025-08-14

**Authors:** Sabrina S Azevedo, Gabriela C Chianca, Bruna A Thurler, Raiane C Chamon, Helvécio C Corrêa Póvoa, Leonardo S Antunes, Natalia L Pontes Póvoa Iorio

**Affiliations:** 1 Experimental and Applied Microbiology Laboratory, Nova Friburgo Institute of Health, Universidade Federal Fluminense, Nova Friburgo, BRA; 2 Department of Pathology, School of Medicine, Universidade Federal Fluminense, Niterói, BRA; 3 Department of Specific Formation, Nova Friburgo Institute of Health, Universidade Federal Fluminense, Nova Friburgo, BRA

**Keywords:** anti-bacterial agents, daptomycin, dental pulp diseases, enterococcus faecalis, root canal medicaments

## Abstract

Introduction

Intracanal medications must have antimicrobial properties, in addition to reaching the natural complexities of the periapical tissues, to help in their recovery. Considering the need for more studies about associations among these medications, we aimed to analyze the antimicrobial activity of daptomycin, alone and associated with Triple Antibiotic Paste (TAP), through the penetrability of bovine dentin against four *Enterococcus faecalis* samples: one standard American Type Culture Collection (ATCC) 29212 sample and three clinical samples.

Methods

Eighty-three bovine freshly extracted teeth were standardized at 15 mm and prepared to file 45, and then sterilized. The roots received, randomly, one of five treatments (*n*=16/group). Group 1: TAP; Group 2: daptomycin; Group 3: TAP+daptomycin; Group 4: calcium hydroxide; and Group 5: propylene glycol. The treated roots were inserted into a previously prepared orifice in the base of Petri dishes, which were, twice a week, filled with solid medium inoculated with *E. faecalis* (five fresh inoculated mediums/15 days). After 24 h incubation with each new inoculum, a reading was performed to determine the presence/absence of inhibition zones around the roots. A total of five readings, on days 1, 4, 8, 11, and 15, were performed for each plate. Three untreated roots with culture medium without inoculum represented the sterility control group.

Results

Group 3 (TAP associated with daptomycin) was the group responsible for the highest number of inhibition zones and the only one capable of inhibiting all *E. faecalis *strains in at least one of the readings. Comparing TAP (group 1) with daptomycin (group 2) alone, group 1 presented a higher number of inhibition zones than group 2. Groups 4 and 5 (calcium hydroxide and propylene glycol) did not present inhibition halos for any of the evaluated *E. faecalis* strains. Absence of microbial growth was observed for all roots in the sterility control group.

Conclusions

This study showed, through the penetrability of bovine dentin, that the association between TAP and daptomycin improved the antimicrobial effectiveness of TAP, demonstrating a possible synergistic activity against *E. faecalis* (ATCC and clinical samples). TAP associated with daptomycin can be considered as a potential intracanal medication, but only as a last resort.

## Introduction

The persistence of apical periodontitis after endodontic treatment is directly related to the persistence of microorganisms that survived the chemomechanical preparation after the use of instruments and irrigating solutions [1]. *Enterococcus faecalis* is one of the main microorganisms associated with endodontic treatment failure [2].

To survive endodontic treatment, bacteria can form biofilm, colonize areas unreachable to root canal instrumentation, persist protected by tissue debris, adapt to new environments through the activation of alternative metabolic pathways or be resistant to the antimicrobial agents used during treatment [1,3]. Intracanal medications are important complementary endodontic tools, being useful mainly in questionable or unfavorable prognosis cases [4]. 

Among the combinations of antibacterial intracanal medications, Triple Antibiotic Paste (TAP), composed of ciprofloxacin, metronidazole, and minocycline, is interesting in endodontics due to its effectiveness against different microorganisms. TAP presents a range of possible applications, such as the regeneration and revascularization of the pulp, also being useful in the cleaning of root canals and healing of periapical lesions [5].

Novel and different combinations and/or proportions of antibacterials composing intracanal medications have been evaluated with the aim of improving the results of endodontic treatments [6]. However, in none of these studies, microbiological analyses have been performed of an intracanal medication containing TAP associated with daptomycin. This cyclic lipopeptide antibiotic with antibiofilm activity [7] is used as a valuable therapeutic option in the treatment of Gram-positive and hard-to-treat infections, such as those caused by *Enterococcus* [8].

The present study aims to evaluate the effectiveness of daptomycin, alone and associated with TAP, in inhibiting *E. faecalis *strains (including endodontic clinical ones) by their ability to penetrate through bovine dentine. The null hypothesis tested was that daptomycin does not increase the effectiveness of TAP.

## Materials and methods

**Figure 1:**
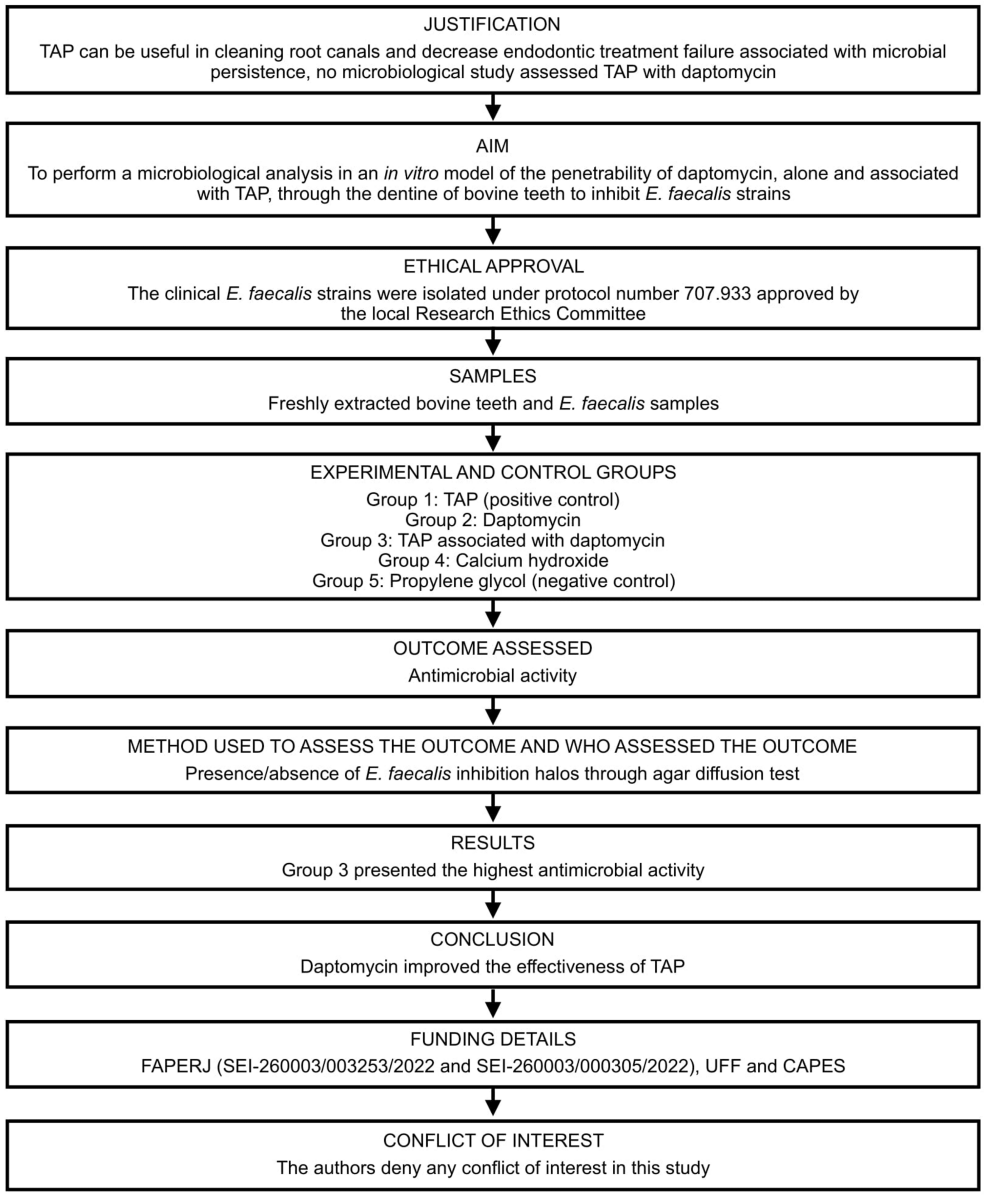
Preferred Reporting Items for Laboratory studies in Endodontology (PRILE) 2021 flowchart presenting the steps involved in this in vitro study. TAP, Triple Antibiotic Paste; FAPERJ, Fundação Carlos Chagas Filho de Amparo à Pesquisa do Estado do Rio de Janeiro; UFF, Universidade Federal Fluminense; CAPES, Coordenação de Aperfeiçoamento de Pessoal de Nível Superior.

This article has been written according to Preferred Reporting Items for Laboratory studies in Endodontology (PRILE) 2021 guidelines [9]. The PRILE 2021 flowchart is presented in Figure 1. This *in vitro* study was performed according to Zaruba and coworkers (2015) [10], with modifications.

Sample size calculation 

The sample size was determined according to a previous study [10] using the area of inhibition halos of *Fusobacterium nucleatum* after 48 h of treatment from the TAP and calcium hydroxide groups. Binomial one-tailed t-test was used in the statistical program BioEstat 5.3 software (Institute for Sustainable Development Mamiraua, Tefe, AM, Brazil, a freeware available at www.mamiraua.org.br) with 5% level of significance and 95% power. Thus, the sample reached a minimum of four roots per experimental group.

Root preparation

A total of 83 freshly extracted bovine teeth, obtained from a slaughterhouse, were included in this study. Teeth that presented cracks in the roots, lateral canals and canals with apical diameter greater than size #30 were excluded. The roots were cut coronally using a water-cooled diamond saw (diamond wafering blade, 0.10 x 22 mm; KG Sorensen, Serra, ES, Brazil) to a final root length of 15 mm. The apical patency was established with a #10 K-File (Dentsply, São Paulo, SP, Brazil) placed up to the apical foramen until the tip was just visible beyond the apex, and 1 mm was subtracted from that patency to define the working length.

The root canal instrumentation was performed using ProTaper® Gold up to F3 (Dentsply) and a K-file #45 (Dentsply) under irrigation with 4 mL 2.5% NaOCl between each file, using 27-gauge lateral irrigation tip (MK Life, Porto Alegre, RS, Brazil), totalizing 24 mL. Then, 5 mL of 17% ethylenediaminetetraacetic acid (EDTA) was used for 5 min to remove the smear layer followed by 10 mL of sterile saline. The roots were then sterilized by autoclaving.

Treatments with intracanal medications

Immediately before treatment, the apical foramen of each root was sealed using a composite resin (FGM, Joinville, Santa Catarina, SC, Brazil) light-cured for 40 s (mode: HIP, 1200 mW/cm^2^, Radii-Cal CX, SDI). The roots were randomly divided into five treatment groups (*n*=16), by drawing (author NLPPI), and treated with one of the following intracanal medications using Lentulo (MK Life), in a laminar flow hood:

Group 1 (G1): TAP (Lenza Farm, Belo Horizonte, MG, Brazil), containing equal parts of ciprofloxacin, metronidazole, and minocycline (positive control); Group 2 (G2): Daptomycin (Eurofarma Laboratórios S.A., Itapevi, SP, Brazil); Group 3 (G3): TAP associated with daptomycin (TAP+Dap), containing equal parts of daptomycin, ciprofloxacin, metronidazole, and minocycline; Group 4 (G4): Calcium hydroxide (Biodinâmica Química e Farmacêutica LTDA, Ibiporã, PR, Brazil); Group 5 (G5): Propylene glycol (Lenza Farm) as negative control.

Groups 1, 2, 3, and 4 were prepared with propylene glycol as a vehicle at a 1:2 (w/v) ratio.

After placement of the intracanal medications, the access was sealed with a dual-curing self-adhesive cement (FGM) light-cured for 40 s (mode: HIP, 1200 mW/cm^2^, Radii-Cal CX, SDI, Boca Raton, FL, USA). After treatment, the roots were labeled with numbers (1-5) (author NLPPI) to conceal each treatment from the reader (author GCC). All root preparations and treatments were performed by the same endodontic specialist (author SSA) to avoid inter-operator variability.

Bacterial inocula 

*E. faecalis* American Type Culture Collection (ATCC) 29212 and three clinical *E. faecalis* strains from root canal infections from our laboratory's culture collection were labeled by letters (A-D) (author NLPPI) and used to prepare four different inocula.

*E. faecalis* strains were kept at -20ºC in Brain Heart Infusion (BHI) broth (Becton, Dickinson and Company, Sparks, MD, USA) with glycerol 20% and transferred onto BHI agar (Becton, Dickinson and Company). After incubation at 36ºC for 24 h, the degree of purity was verified and isolated bacterial colonies of each strain were transferred to 0.9% sterile saline reaching an optical density of 1.00±0.05 nm (Libra S2 Colorimeter, Biochrom, Cambridge, England) corresponding to an inoculum with approximately 10^9^ colony-forming units per milliliter (CFU/mL).

Agar diffusion test

**Figure 2:**
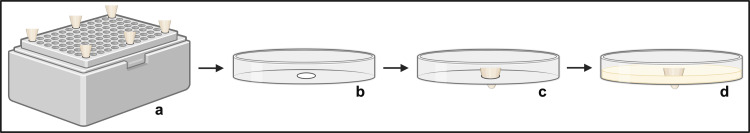
Preparation of set (treated root and Petri dish). (a) Teeth treated with intracanal medications; (b) Petri dish with orifice in its base; (c) set (root inserted in the orifice of the Petri dish base); (d) set with fresh medium+inoculum. (Created with BioRender.com/zj9lt32; BioRender, Ontario, Canada).

After treatment, the roots were inserted in the base of a sterile Petri dish (60 mmx15 mm) through an orifice previously prepared. The free space between the root and this orifice as well as the exposed apical portion (3 mm) of the root were completely sealed with sticky wax (Asfer Industria Quimica ltda, São Caetano do Sul, SP, Brazil) (Figure 2).

Each set (treated root and Petri dish) was submitted to another randomization, by drawing (author SSA), in order to define which *E. faecalis* strain would be inoculated in each dish. 

**Figure 3:**
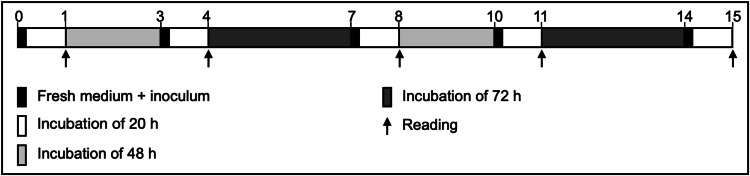
Assay timeline from day 0 to 15.

Thereafter, 350 µL of each inoculum was added to a tube containing 12 mL of sterile BHI agar (Becton, Dickinson and Company) at 55ºC, homogenized and placed in each Petri dish (Figure 2). After agar solidification, the sets were aerobically incubated at 36°C for 15 days. The inoculated BHI agar was replaced twice a week, totalizing five fresh inoculated mediums in the 15-day period. After the 24 h incubation period with the new inoculated medium, each set was submitted to a reading to determine the presence/absence of inhibition halos. A total of five readings, on days 1, 4, 8, 11 and 15, were performed for each Petri dish (Figure 3). The reader (author GCC) was blinded to the treatment and the *E. faecalis* strain used. Three extra roots were used as sterility control, receiving medium without inoculum, and had their apical foramen and access sealed.

Statistical analysis

Data were statistically analyzed using GraphPad Prism (version 6.0, Dotmatics, Boston, MA, USA) software, with a 95% level of confidence. Comparative analyses between the results from the number of inhibition halos of *E. faecalis* strains along the experimental days and number of times (out of five readings) halos were produced by each root were performed by two-way analysis of variance (ANOVA) followed by Tukey’s test. Statistical significance was considered when *p* values <0.05 were obtained.

## Results

**Figure 4:**
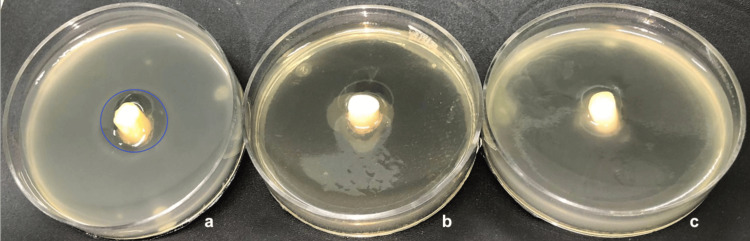
Representative picture of presence/absence of E. faecalis inhibition halos. (a) Presence of *E. faecalis* inhibition halo by TAP+Dap (highlighted in blue); (b) sterility control (medium without inoculum); (c) absence of *E. faecalis* inhibition halo (negative control) (G5). The shadow observed in image b is due to the sticky wax used to seal the free space between the root and the base of the Petri dish.

**Figure 5:**
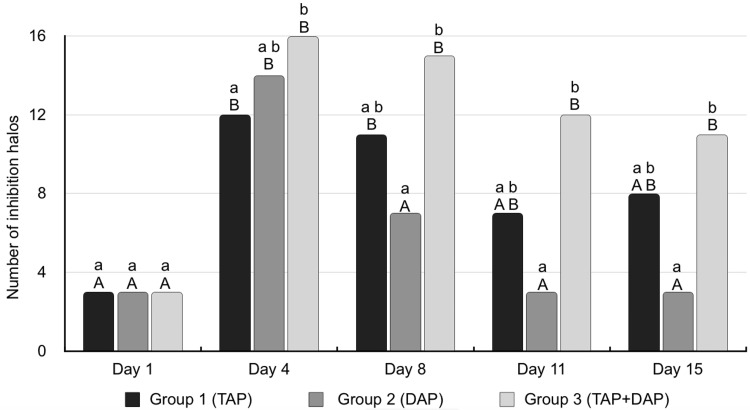
Number of inhibition halos of E. faecalis strains along the experimental days. TAP, Triple Antibiotic Paste;  DAP, daptomycin; TAP+DAP, TAP associated with daptomycin; Different lowercase letters in the same day represent statistically significant results between groups (*p*<0.05), different uppercase letters in the same group of treatment represent statistically significant results between analyzed days (*p*<0.05). G4 and G5 did not inhibit any *E. faecalis* strains and are therefore not depicted in this figure.

Eighty-three bovine teeth roots were included in this study (16 per group, plus three extra roots used as sterility control) in order to evaluate the penetrability of intracanal medications in inhibiting *E. faecalis* growth (Figure 4). The presence of inhibition halos around the roots of the four *E. faecalis* strains tested against the five treatment groups after 1, 4, 8, 11, and 15 days of treatment is presented in Figure 5.

The association between TAP and daptomycin (G3) was the only treatment at a given moment that was able to inhibit all *E. faecalis* strains; and this was only observed on day 4. Group 3 remained responsible for the greatest number of inhibition halos on the subsequent days. Significant differences in halo inhibitions were observed on day 4 (between G3 and G1; *p*=0.0337), and on days 8 (*p*=0.0014), 11 (*p*=0.0008) and 15 (*p*=0.0033) between G3 and G2 (Figure 5).

All roots from groups 4 (calcium hydroxide) and 5 (propylene glycol) did not inhibit any of the evaluated *E. faecalis* strains. These groups did not present significant differences between each other during any of the experimental days. Additionally, no difference was observed between these groups (4 and 5) and any of the other groups on day 1, nor with daptomycin (G2) on days 11 and 15.

Day 4 was the moment when all antibiotics (alone or combined) had the greatest number of halos, whereas day 1 presented the lowest, showing significant differences between both moments for all antibiotics (G1-G3) (Figure 5).

Group 1 (TAP) activity through the dentine still remained high on day 8, compared with day 1 (*p*<0.0001). After group 2 (daptomycin) achieved its highest activity (day 4), there was a drop on the following days, behaving similarly to day 1 (*p*≥0.1355). On the other hand, TAP+Dap (G3) was the only treatment that remained responsible for a high number of inhibition halos from day 4 until the end of this experiment (day 15) (Figure 5).

Each treatment group (G1-G5) containing four roots per *E. faecalis* strain was read at five different time-points (days 1, 4, 8, 11, and 15). Table 1 shows how many times halos were produced by each treated root during these five readings. The association between TAP and daptomycin (G3) presented more halos per root along the five readings, resulting in the highest means (indicated in Table 1 by the * symbol), and it was the only treatment that showed statistical differences when compared to groups 4 and 5 for all *E. faecalis* strains.

**Table 1:** Number of times (out of five readings) halos were produced by each treated root TAP, Triple Antibiotic Paste (ciprofloxacin, metronidazole and minocycline); G, group; ATCC, American Type Culture Collection; #, number; SD, Standard Deviation; -, absence of halo. Different letters in the same column represent statistically significant results between treatment groups (p<0.05). *, only treatment that showed statistical differences when compared to groups 4 and 5 for all *E. faecalis* strains. Each column under each bacterial strain represents, per root, the number of days (out of five reading days) when a halo was observed. An extra column shows their means.

Intracanal Medications	ATCC 29212					Clinical strain 1					Clinical strain 2					Clinical strain 3				
	# of times halos/root				Mean (SD)	# of times halos/root				Mean (SD)	# of times halos/root				Mean (SD)	# of times halos/root				Mean (SD)
TAP (G1)	4	1	-	4	2.25 (2.06)^a,b^	4	3	4	3	3.5 (0.58)^a^	2	-	4	1	1.75 (1.71)^a,b^	4	1	2	4	2.75 (1.5)^a,b^
Daptomycin (G2)	2	2	1	4	2.25 (2.06)^a,b^	-	1	1	3	1.25 (1.26)^b^	2	1	1	2	1.5 (0.58)^a,b^	2	1	2	5	2.5 (1.73)^b,c^
TAP + daptomycin (G3)	4	3	2	4	*3.25 (0.96)^a^	5	4	3	4	*4 (0.82)^a^	3	1	4	4	*3 (1.41)^a^	4	3	4	5	*4 (0.82)^a,b^
Calcium hydroxide (G4)	-	-	-	-	0^b^	-	-	-	-	0^b^	-	-	-	-	0^b^	-	-	-	-	0^c^
Propylene glycol (G5)	-	-	-	-	0^b^	-	-	-	-	0^b^	-	-	-	-	0^b^	-	-	-	-	0^c^

The mean number of halos for the clinical samples during these five readings were different from each other as well as when compared to the ATCC *E. faecalis* strain (Table 1). Compared to ATCC, clinical strain 2 showed the lower means for all groups containing antimicrobial substances (G1-G3) and clinical strain 3, higher. Clinical strain 1, on the other hand, did not demonstrate a clear increase/decrease in the mean number of halos for groups G1-G3.

The three sterility control plates showed absence of bacterial growth during all the experimental readings.

## Discussion

Endodontic treatment failure can be related to several factors, such as bacterial persistence in planktonic form or as biofilms in the root canal system [1,11,12]. In these cases, microorganisms can be located intra- and extra-radicular [11] as well as within dentinal tubules [13]. The use of intracanal medications, commonly calcium hydroxide and antibiotic preparations, help eliminate remaining bacteria within thewh root canal system [14], including in endodontic retreatment [2]. To the best of our knowledge, this is the first laboratory study to evaluate the penetrability of an association between TAP and daptomycin through the root dentine of bovine teeth to inhibit* E. faecalis* samples.

We chose *E. faecalis* as the inoculum in this study due to its ability to form biofilms, escape disinfection measures, resist several antimicrobials [3], tolerate high pH levels [15], and its major role in persistent periradicular lesions after failure of endodontic treatment [2,3].

Propylene glycol, used as the vehicle in groups 1-4, increases the speed of delivery of intracanal medications through the dentine and, as a viscous vehicle, is able to maintain the intracanal medication in the contact areas for longer periods of time [16]. Calcium hydroxide has high alkalinity, antibacterial activity, ability to neutralize endotoxins and to dissolve tissues [4]. The combination of these substances, represented by G4 of this study, is widely used as an intracanal medication [5]. Our results showed no inhibition of any of the evaluated *E. faecalis* strains by G4, which can be explained by the high pH tolerance of this species [15], enduring values as high as 11 [17], and therefore persisting in this environment. Similar *in vitro*results were found by Zaruba and colleagues (2015) [10], who evaluated a mixture of calcium hydroxide and saline against* Fusobacterium nucleatum*. Although our study did not find antimicrobial activity for calcium hydroxide, this substance has been demonstrated to be effective against several common endodontic pathogens, being exceptionally less effective against *E. faecalis* (microorganism used in our study) and *Candida albicans* [18].

Groups 1, 2, and 3, containing antimicrobial substances and propylene glycol as a vehicle were able to inhibit the growth of all *E. faecalis* strains in at least one of the evaluated moments. Some of these halos did not present regular circular shapes, which might be explained by the heterogeneous development of sclerotic dentine in certain areas of the root [19] and this could, in turn, alter its penetrability to the herein analyzed antimicrobial substances [20].

An increase in the number of halos was observed between days 1 and 4 of this study, and day 4 was the moment in which all groups containing antibiotics (G1-G3) presented the greatest number of halos. On the first day, we believe the antibiotics had possibly not yet penetrated through the full length of the dentinal tubules, therefore not reaching enough concentration on the external root surface to inhibit microbial growth. In contrast, on the following reading (day 4), the halos observed in many (87.5%) of the samples could indicate that the antibiotics were able to penetrate through the tubules. In the subsequent days of this experiment, the antibiotic activity decreased (being observed by a lower number of halos), likely due to the fact that after antibiotic reconstitution, a decrease in effectivity is observed over time, especially if stored at high temperatures [21], such as the incubation temperature we used. Although aware of these properties, we have decided on a two-week following, since one to two weeks is the common interval between endodontic sessions [22].

Furthermore, the viscosity of the vehicle that makes up the intracanal medications determines the speed of penetration and absorption in the root canal. Aqueous vehicles are more soluble and are resorbed quicker, remaining less time in contact with the diseased tissue. Viscous vehicles, on the other hand, maintain the intracanal medications longer in the canal, which prolongs their antimicrobial activity [23]. We observed a decrease in antimicrobial activity after day 4, which might also be attributed to the viscosity of the vehicle used. Although propylene glycol is a viscous vehicle, other vehicles, such as glycerine, have higher viscosity and could have prolonged the antimicrobial effect of the intracanal medications.

The molecular weight of a substance is correlated with its rate of diffusion through dentine. The molecular weight of daptomycin (1,619.709 g/mol) is higher than that of the other substances tested (calcium hydroxide (74.093 g/mol), ciprofloxacin (331.346 g/mol), metronidazole (171.16 g/mol), propylene glycol (76.09 g/mol), and minocycline (457.4764 g/mol)). However, the observation of the diffusion halo confirms its ability to penetrate dental canals and diffuse through agar, effectively inhibiting microbial growth.

Comparing TAP alone (G1) or associated with daptomycin (G3), we could observe that G3 was responsible for a higher mean number of days in which the treatments inhibited the growth of *E. faecalis* per root. After day 1, G3 also demonstrated a higher number of inhibition halos per day than G1. This difference was even more noticeable after day 4, where the values showed statistical differences between G1 (TAP) and G3 (TAP+Dap), therefore rejecting the null hypothesis.

The *E. faecalis* strains evaluated in this study demonstrated different behaviors against the antimicrobials tested, which was expected since clinical and ATCC strains respond differently to antimicrobial agents [24].

Synergism between antibacterial agents is a widely known phenomenon, being an option in treating multidrug-resistant infections. Many combinations and/or proportions of antibacterial substances have been proposed to treat microbial infections [25], including those containing daptomycin against *E. faecalis* [26]. So, in this study, we might attribute the better performance of G3 (TAP+Dap) to this phenomenon.

Daptomycin is a cyclic lipopeptide antibiotic primarily used to treat severe infections, including complicated skin and soft tissue infections, right-sided infective endocarditis, bloodstream infections, meningitis, sepsis, and urinary tract infections, caused by Gram-positive pathogens, such as enterococci [7]. It is particularly effective against this bacterial gender and is often considered a last-resort option for multidrug-resistant *E. faecium* infections [27]. So, even though a possible synergistic activity was observed in this study, daptomycin use should be cautious and reserved for severe infections as a last resort to treat complicated endodontic infections, such as successive failures in endodontic treatment.

A limitation of our study is that we used bovine instead of human roots and controversial data exist regarding dentine microleakage in bovine when compared to human roots [28,29]. According to Tanaka and coworkers, the structural differences between bovine and human teeth are reflected in their respective radiographic densities, due to the different constitution and mineral content of bovine and human teeth. Even though these authors found statistical differences between bovine and human coronal dentin radiodensity, no statistically significant differences between bovine and human radicular dentin were observed [30].

Even with this limitation, *in vitro* studies using bovine teeth are widely used in endodontic research, probably to avoid limiting factors associated with the use of human teeth, such as the difficulty in obtaining the number of human teeth needed for performing laboratory assays [31]. Furthermore, since the chemical composition of human enamel and dentine showed similarity to bovine enamel and dentine, bovine teeth should be the first choice as substitutes for human teeth in research [32].

We believe that future studies evaluating the biocompatibility of TAP associated with daptomycin, its action against endodontic biofilm models, and different viscous vehicles might complement and improve our findings.

## Conclusions

In this study, we investigated the effectiveness of daptomycin, alone and associated with TAP, in inhibiting *E. faecalis* strains by their ability to penetrate through bovine dentine. We showed that daptomycin improved the antimicrobial effectiveness of TAP, demonstrating a possible synergistic activity against *E. faecalis*. TAP associated with daptomycin has promising results for endodontic treatment as an alternative intracanal medication; however, daptomycin use should be cautious and reserved for severe infections as a last resort. 
